# ER-positive and *BRCA2*-mutated breast cancer: a literature review

**DOI:** 10.1186/s40001-023-01618-1

**Published:** 2024-01-06

**Authors:** Pu-Chun Li, Yi-Fan Zhu, Wen-Ming Cao, Bei Li

**Affiliations:** 1 Postgraduate Training Base Alliance of Wenzhou Medical University (Zhejiang Cancer Hospital), Hangzhou, 310022 China; 2 Department of Breast Medical Oncology, Zhejiang Cancer Hospital, Hangzhou, 310022 China; 3 Department of Geriatric, Affiliated Hangzhou First People’s Hospital, School of Medicine, Westlake University, Hangzhou, 310006 China

**Keywords:** Breast cancer, *BRCA2* mutation, ER-positive, PARP inhibitor, CDK4/6 inhibitor, Survival rate

## Abstract

*BRCA2*-mutated carriers have a high lifetime risk of breast cancer (BC), an early age of onset, and an increased risk of other cancers (including ovarian, pancreatic, and prostate cancer). Almost 70–80% of *BRCA2*-mutated BC are estrogen receptor (ER)-positive, which is a particular type of ER-positive BC that differs from sporadic ER-positive BC. This article reviews the clinicopathological features, treatment, and prognosis of ER-positive and *BRCA2*-mutated BC to provide a reference for clinical decision-making.

## Introduction

The most prevalent malignancy and the main reason for cancer-related mortality in women globally is BC [1]. It has been established that the development of BC is mainly the result of a multifactorial interaction of genetic, environmental, and lifestyle factors. *BRCA2* is one of the most prevalent BC susceptibility gene. The lifetime risk of developing BC in germline *BRCA2*-mutated carriers is approximately 55%, significantly higher than non-carriers [2]. However, the difference in prognosis between *BRCA2*-mutated and non-mutated BC is still controversial [3]. One study found that the majority of females with a *BRCA2* mutation have ER-positive and human epidermal receptor 2 (HER2)-negative BC [4]. Among sporadic BC, ER-positive BC are often characterized by late age of onset, low pathological grade, and low rate of lymph node metastasis and have a significantly better prognosis than do ER-negative BC [5, 6]. However, studies have shown that the risk of death is higher in ER-positive than in ER-negative BC with a *BRCA2* mutation and that ER positivity is an independent prognostic factor [7].

Endocrine therapy is the primary treatment modality for ER-positive BC, and in combination with cyclin-dependent kinases 4 and 6 (CDK4/6) inhibitors is currently the first-line therapy for ER-positive metastatic BC [8–10]. Moreover, abemaciclib combined with endocrine therapy enhanced invasive disease-free survival (iDFS) in ER-positive patients with high risk of recurrence [11]. However, it has been found that germline *BRCA2*-mutated BC are often associated with copy number loss of the *Rb1* gene, leading to CDK4/6 inhibitors resistance [12]. Therefore, the optimal therapy strategy for ER-positive, *BRCA2*-mutated BC is currently unclear. In addition, the *BRCA2* mutation causes homologous recombination repair defects and may be more susceptible to DNA damage drugs such as poly (adenosine diphosphate-ribose) polymerase (PARP) inhibitors and platinum. PARP inhibitors combined with immune checkpoint inhibitors (ICIs) have also demonstrated favorable performance in the management of ER-positive and *BRCA2*-mutated BC [13]. The regulatory relationship between ER and BRCA2 should be investigated to elucidate the internal features and clinical phenotypes of ER-positive BC with *BRCA2* mutation and to advance treatment research.

This article reviews the clinicopathological features, treatment, and outcomes of ER-positive and *BRCA2*-mutated BC.

## Interaction between ER signaling pathway and BRCA2

Located in 13q12.3, the *BRCA2* gene is involved in cell cycle regulation, signal transduction, and DNA damage repair through the Rad51 binding region [14, 15]. The most important function of the BRCA2 protein is participation in damage repair of double-stranded DNA via homologous recombination pathway [16]. However, the exact carcinogenic mechanism and the factors that regulate the risk of BC are still unknown. *BRCA2*-mutated BC mainly present as ER-positive and HER2-negative, suggesting that there may be an intrinsic relationship between the ER signaling pathway and BRCA2.

**Fig. 1:**
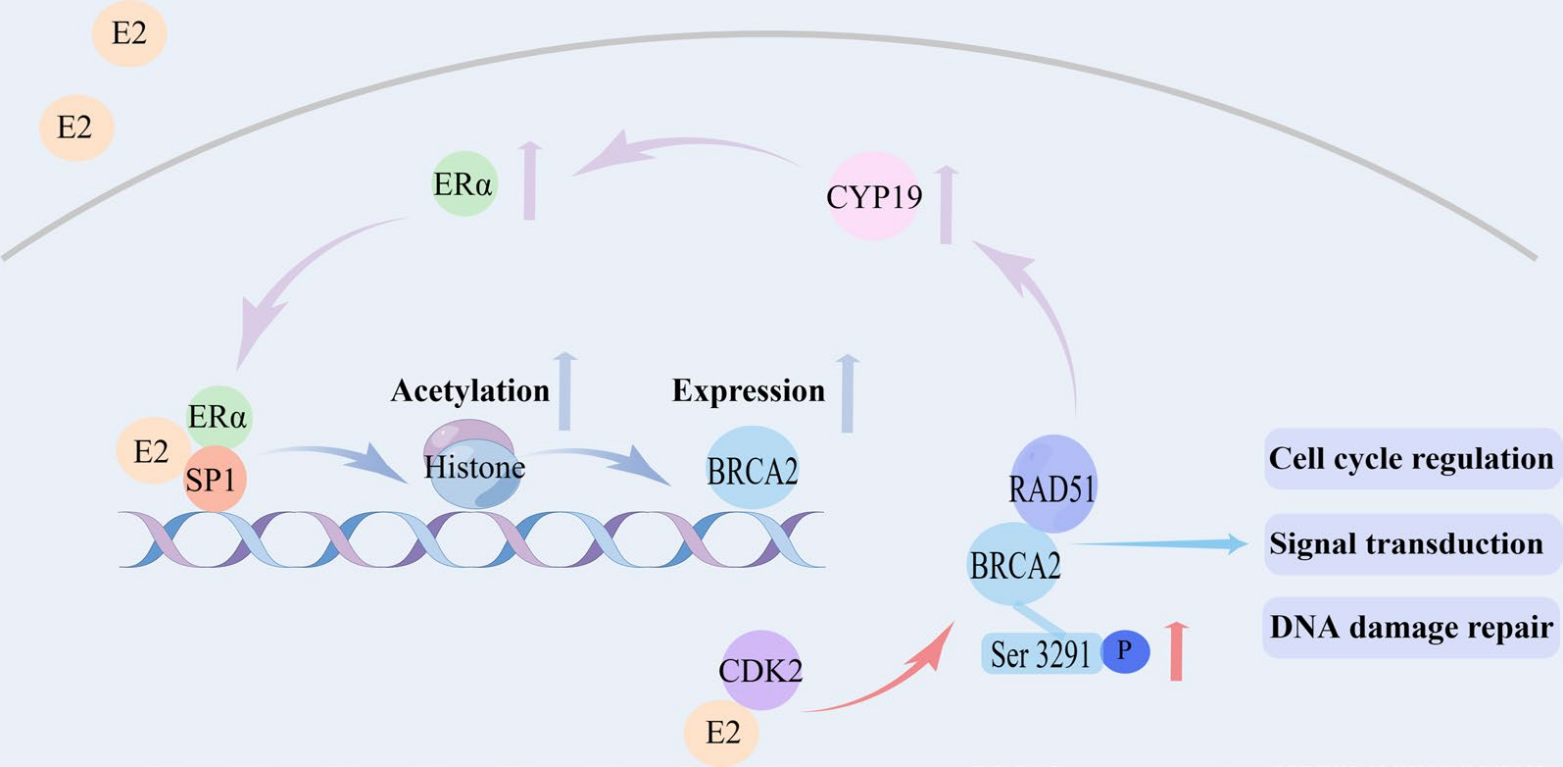
Interaction between ER signaling pathway and BRCA2

Estradiol (E2) acts through the nuclear receptor ERα or ERβ, and E2 binds to ERα to form an activating transcription complex, which binds to sequence-specific DNA binding proteins 1 (SP1), inducing histone acetylation, to activate *BRCA2* transcription [17]. Ser3291 is located at the C-terminus of BRCA2, and the loss of phosphorylation of Ser3291 may eliminate the tumor suppressor function of BRCA2. E2 rapidly phosphorylates Ser3291 in a CDK2-dependent manner, thereby stabilizing BRCA2 protein and enhancing the DNA damage repair response of cells [18]. These results indicate that E2 can positively regulate BRCA2 (Fig. 1).

In addition, many somatic mutations in the *BRCA2* gene are observed among ER-positive BC. E2 promotes the rapid proliferation of cancer cells through the ER signaling pathway, along with the increase of DNA replication and damage of cancer cells (indirect mutagenesis effect) and the action of genotoxic metabolites (direct mutagenesis effect). Then, the *BRCA2* gene starts to mutate or be lost, leading to functional inactivation and resulting in loss of homologous recombination repair function [19].

Normal BRCA proteins can regulate and induce appropriate expression and transcriptional activity of ER through the *CYP19* gene. It can also promote estrogen signaling through appropriate estrogen synthesis. In contrast, *BRCA* gene mutation that leads to impaired BRCA protein function is related to defects in both estrogen signaling and DNA repair [20]. The high risk of premature ovarian failure in *BRCA2*-mutated BC reflects altered estrogen signaling [21].

## Prevention of *BRCA2*-mutated carriers

### Detection and surveillance

In healthy women with a *BRCA2* mutation, the lifetime risk of developing BC and ovarian cancer (OC) ranges from 38.0% to 84.0% and 16.5% to 27.0%, respectively [2, 22–24]. Consequently, it is essential to conduct regular surveillance for BC and OC. Screening for BC in healthy women with a *BRCA2* mutation should commence with monthly self-examinations at the age of 18. After reaching 25 years, individuals should undergo biannual or annual breast examinations by a physician. Annual breast magnetic resonance imaging or mammography is recommended for women aged 25 to 30, and both modalities are recommended for women over 30 years old [25–27]. At present, there is a lack of reliable screening modalities for the diagnosis of OC, with diagnosis primarily relying on vaginal sonography and CA125 blood testing [28].

### Prophylactic surgery

Regarding the susceptibility of BC and OC, *BRCA2*-mutated carriers are advised to consider prophylactic bilateral mastectomy (PBM) and prophylactic bilateral salpingo-oophorectomy (PBSO). While PBM has been shown to reduce the incidence of BC by approximately 90% [29–31], it has been demonstrated that there is no survival benefit for *BRCA1/2*-mutated carriers [32]. PBSO is associated with a reduced risk of BC and OC, as well as a decreased risk of BC mortality in *BRCA1/2*-mutated carriers. A meta-analysis revealed that PBSO resulted in a 53% reduction in the incidence of BC among *BRCA2*-mutated carriers and a 79% reduction in the incidence of OC among *BRCA1/2*-mutated carriers [33]. A prospective study reported that PBSO resulted in an 82% reduction in the incidence of OC in *BRCA2*-mutated carriers [34]. Based on the above findings, it is advisable to consider PBSO in *BRCA2*-mutated carriers after childbearing desire completion. Moreover, the administration of short-term hormone replacement therapy following PBSO does not appear to have an impact on the risk of BC in *BRCA1/2*-mutated carriers [35]. However, further research is necessary to substantiate this finding. Patients with a *BRCA2* mutation exhibit a 26% likelihood of developing contralateral breast cancer (CBC) within 20 years following their initial BC diagnosis [2]. A study involving 390 stage I–II BC patients with *BRCA1/2* mutations demonstrated that women who received contralateral prophylactic mastectomy experienced a 48% reduction in the risk of mortality compared to those who underwent unilateral mastectomy [36]. Consequently, it is justifiable to propose bilateral mastectomy as an early intervention for *BRCA2*-mutated BC.

### Chemoprevention

The NSABP-P1 breast cancer prevention trial showed that tamoxifen reduced the risk of developing BC by 62% in *BRCA2*-mutated carriers (RR = 0.38, 95% CI 0.06–1.56) [37]. Duffy et al. demonstrated a 27% reduction in BC risk among *BRCA2*-mutated carriers treated with tamoxifen [38]. In addition, tamoxifen can also reduce the risk of developing CBC by 37%-58% in *BRCA2*-mutated carriers [39, 40]. The STAR study compared the relative effects of raloxifene and tamoxifen in preventing invasive BC [41]. This trial initially demonstrated that the effectiveness of raloxifene was similar to tamoxifen in preventing invasive BC. An updated analysis with a median follow-up of 81 months from this trial revealed that raloxifene was as effective as tamoxifen in preventing invasive BC, with 76% of the effectiveness, and demonstrated lower toxicity, including significantly fewer cases of endometrial cancer and thromboembolic events, compared to tamoxifen [42]. The FDA has approved tamoxifen and raloxifene for BC prevention in high-risk women. It has been suggested that aromatase inhibitors (AIs) reduce the risk of CBC in *BRCA1/2*-mutated BC [43]. There is no research exploring the preventive effect of AIs for *BRCA2*-mutated carriers.

## Clinicopathological features and prognosis of *BRCA2*-mutated BC

### Clinicopathological features

The clinicopathological features associated with BC prognosis include tumor diameter, lymph node metastasis, and pathological grade [44]. *BRCA2*-mutated patients have a higher pathological grade and a higher frequency of positive lymph nodes than those without the mutation [45, 46]. Jonasson et al. [47] retrospectively studied the clinicopathological features of 285 cases of BC with *BRCA2* c.999del5 mutation and 570 cases without mutation. They found that compared with those without mutation, those with *BRCA2* mutation had significantly larger tumor diameter (2.7 cm vs 2.4 cm, *P* < 0.001), higher lymph node-positive rate (55% vs 43%, *P* = 0.001), and higher incidence of second or contralateral BC (18.6% vs 6.7%, *P* < 0.001). ER positivity in *BRCA2*-mutated BC was strongly associated with lymph node metastasis and tumor diameter but not with pathological grade.

Olafsdottir et al. [48] evaluated 608 patients with *BRCA2*-mutated BC and found that the rate of lymph node positivity was apparently higher in ER-positive individuals than in ER-negative individuals (59% vs 34%, P < 0.001). They also indicated that the proportion of ER positivity in *BRCA2*-mutated BC decreased with increasing age at onset (≤ 39 years: 83%; 40–50 years: 79%; > 50 years: 72%; *P* = 0.01). Evans et al. [49] analyzed 664 cases of stage I–III BC with a *BRCA2* mutation in 5 databases and found consistent results that ER-positive tumor rates decreased as the age of onset decreased: < 40 years, 83%; 50–60 years, 79%; and ≥ 60 years, 76%. Among sporadic BC, the proportion of ER-positive tumors increased with age [50]. Early-onset of ER-positive BC in *BRCA2*-mutated carriers may be associated with high basal E2 levels [51].

### Prognosis

After adjusting for other prognostic factors and therapy, Jonasson et al. [47] demonstrated that patients with ER-positive BC with a *BRCA2* mutation had worse prognosis than did patients with ER-negative BC(HR = 1.61, 95% CI 1.11–2.35, *P* = 0.01). Metcalfe et al. [52] evaluated 390 *BRCA2*-mutated BC individuals identified between 1975 and 2015 and found that 77% of them were ER-positive. According to multivariate analysis, ER positivity was an independent prognostic factor and ER-positive patients had a higher risk of death than had ER-negative patients (HR = 2.08, 95% CI 0.99–4.36, *P* < 0.05). The 10- and 20 year survival rates for *BRCA2*-mutated, ER-positive patients were 80.4% and 62.2%, respectively, significantly lower than the 92.6% and 83.7% rates for ER-negative patients (*P* = 0.03). Vocka et al. [53] followed 191 *BRCA1/2*-mutated BC patients (151 *BRCA1*-mutated and 40 *BRCA2*-mutated) and 680 non-mutated patients. They found that among the patients with ER-positive BC, the 10-year disease-free survival (DFS) and 10-year disease-specific survival (DSS) of the *BRCA2*-mutated patients were significantly lower than those of the non-mutated patients. Multigene expression profiling models are commonly used to evaluate prognosis and formulate treatment strategies for early BC. Oncotype DX quantifies the likelihood of relapse of ER-positive BC and the potential benefit of chemotherapy [54]. A retrospective study compared the distribution of Oncotype DX recurrence risk scores between 143 *BRCA1/2*-mutated, ER-positive BC and 1594 sporadic BC [55]. The results revealed that the number of patients with a high recurrence risk of ER-positive and *BRCA1/2*-mutated BC was approximately 3 times higher than patients with sporadic BC (*P* < 0.002). Further, the decision for chemotherapy based on recurrence risk score was similar between the mutation group and the non-mutation group. Based on the above results, we can conclude that ER-positive BC have a worse prognosis than ER-negative BC in *BRCA2*-mutated patients and *BRCA2*-mutated BC have a worse prognosis than non-*BRCA2*-mutated BC in ER-positive patients.

In conclusion, *BRCA2*-mutated BC are more common in ER-positive patients and have a worse prognosis than non-mutated ER-positive patients. This is possibly because E2 induces DNA double-strand breaks (DSB) through ERα. The BRCA2 is essential for DNA double-strand damage repair [56, 57]. Higher levels of E2 and ERα further aggravate the degree of DSB in *BRCA2*-mutated tumors, leading to a worse prognosis of BC.

## Surgical treatment

A meta-analysis of 23 studies with 2200 *BRCA1*-mutated and 1212 *BRCA2*-mutated BC found that compared with mastectomy, breast-conserving surgery (BCS) was associated with a higher risk of local recurrence (HR = 4.54, 95% CI 2.77–7.42, *P* < 0.05) [58]. Meanwhile, there was no significant difference in the risk of death (HR = 1.10, 95% CI 0.72–1.69, *P* < 0.001) between the two modalities.

The rate of ipsilateral breast tumor recurrence (IBTR) after BCS for *BRCA1/2*-mutated BC is significantly higher than that for non-mutated BC [59, 60]. Cao et al. retrospectively analyzed whether mutation status affects IBTR after BCS among Chinese patients [61]. They included 103 *BRCA1/2*-mutated BC and 1844 non-mutated BC in Chinese patients. Within a median follow-up time of 80 months, there was no significant difference in IBTR between *BRCA1/2*-mutated BC and non-mutated BC (3.9% vs 2.0%, *P *= 0.16). When IBTR was further divided into new primary tumor (NP) and true local recurrence (TR), the incidence of NP was significantly higher in *BRCA1/2*-mutated BC than in non-mutated BC (3.9% vs 0.6%, *P* < 0.001). Another cohort study compared survival after breast-conserving therapy (BCT) and total mastectomy between *BRCA1*/2-mutated BC and non-mutated BC, with 8396 Chinese BC patients (187 *BRCA1*-mutated, 304 *BRCA2*-mutated, and 7905 non-mutated) included in the study [62]. Within a median follow-up time of 7.5 years, breast cancer-specific survival (BCSS) and overall survival (OS) did not differ significantly between *BRCA1/2*-mutated BC treated with BCT and those treated with either total mastectomy plus radiotherapy or total mastectomy alone (*BRCA2*: BCSS, HR = 0.46, P = 0.17; OS: HR = 0.72, *P* = 0.52).

Although there is an increased incidence of NP after BCS in *BRCA1/2*-mutated BC, there is no difference in OS between BCS and mastectomy. In addition, BCS can improve quality of life, and thus, the guidelines recommend BCS as a relative contraindication for *BRCA1/2*-mutated BC.

## Adjuvant therapy

### Adjuvant chemotherapy

Jonasson et al. [47] found a 15 year specific survival rates of 55% and 75% for *BRCA2*-mutated and non-mutated BC, respectively. Patients with a *BRCA2* mutation who received adjuvant chemotherapy (included anthracycline and non-anthracycline) had a longer DSS than those who did not (HR = 0.35, 95% CI 0.16–0.80, *P* = 0.01). Among patients who did not receive adjuvant chemotherapy, the mutation group had worse prognosis than had the non-mutation group (HR = 2.38, *P* = 0.005). Meanwhile, in patients who received adjuvant chemotherapy, the mutation group had similar prognosis to the non-mutation group (HR = 1.21, 95% CI 0.74–2.00, *P* = 0.5). Olafsdottir et al. [48] showed that in *BRCA2*-mutated patients, the 20 year DSS of those who received adjuvant chemotherapy (the majority received an anthracycline) was better than that of those who did not receive adjuvant chemotherapy (HR = 0.65, 95% CI 0.43–1.00, *P* = 0.05).

Preclinical studies have proved that *BRCA*-mutated BC cell lines are susceptible to DNA-damaging drugs including anthracyclines and platinum but not to taxanes [63]. Platinum is often added to the adjuvant regimen in clinical practice. However, in the current guidelines, the adjuvant chemotherapy regimen including taxanes, anthracyclines, and cyclophosphamide for ER-positive and *BRCA1/2*-mutated individuals is still the same as that for sporadic individuals owing to the lack of large-scale evidence.

### Adjuvant endocrine therapy

Adjuvant endocrine therapy can significantly reduce the risk of recurrence, metastasis, and death in ER-positive BC [64]. Nevertheless, the efficacy of endocrine therapy in *BRCA2*-mutated BC is still controversial. In a prospective study involving 71 *BRCA2*-mutated BC patients and 1550 sporadic BC patients, adjuvant endocrine treatment did not reduce the risk of death in the *BRCA2*-mutated group (HR = 2.05, 95% CI 1.07–3.91, P = 0.03) [65]. Tamoxifen also did not reduce the risk of death in patients with ER-positive, *BRCA2*-mutated BC (HR = 0.91, 95% CI  0.49–1.69, P = 0.76) [52]. Evans et al. [49] also reported that tamoxifen or AIs had no benefit on 10 year survival in ER-positive, *BRCA2*-mutated patients (ER-positive vs ER-negative: 78.9% vs 82.3%, HR = 1.48, 95% CI 0.69–3.20, *P* = 0.31). This may be due to the fact that tumors were more aggressive in patients receiving endocrine therapy than in non-recipients. Meanwhile, individuals who underwent bilateral oophorectomy had a significantly higher 10-year survival rate than had non-recipients (89.1% vs 59.0%; HR = 0.45, 95% CI 0.28–0.72, *P* = 0.001). This similarly reflects abnormalities in ER signaling in *BRCA2*-mutated BC.

Olafsdottir et al. [48] also reported that in *BRCA2*-mutated BC, the relationship between ER status and survival varied according to ovarian resection or endocrine therapy. Patients with ER-positive BC who underwent oophorectomy had a longer 5 year DSS than did those with ER-negative BC (HR = 0.03, 95% CI  0.00–0.29, *P* < 0.01). At 5 years, ER-positive patients who underwent oophorectomy had a 39% lower risk of death than ER-negative individuals (HR = 0.03, 95% CI 0.00–0.29, *P* < 0.01). Meanwhile, ER-positive patients who did not undergo oophorectomy had a significantly higher risk of death than ER-negative patients (HR = 1.99, 95% CI 1.11–3.59, *P* = 0.02). Similarly, ER-positive patients who received endocrine therapy had a 28% lower risk of death than ER-negative patients (HR = 0.72, 95% CI 0.32–1.61, P = 0.43), and those who did not receive endocrine therapy had a worse prognosis (HR = 2.36, 95% CI 1.26–4.44, *P* = 0.01).

Clinically, the decision for adjuvant chemotherapy for ER-positive, *BRCA2*-mutated BC remains stratified according to risk factors for recurrence. It is currently unclear whether tamoxifen or AIs improve the prognosis of ER-positive and *BRCA2*-mutated BC. However, in clinical practice, endocrine therapy is routinely used after surgery for ER-positive patients. Bilateral oophorectomy has significant benefits, but ovarian function inhibitors may be considered as an alternative for women with fertility needs.

## PARP inhibitor therapy

*BRCA*-inactivating mutations cause defects in homologous recombination repair in tumor cells, leaving these cells highly dependent on the single-strand break repair pathway. This pathway is regulated by PARP, and PARP inhibitors cause cell death by accumulating irreparable DNA damage [66]. A growing number of phase III clinical trials is showing the efficacy of PARP inhibitors in *BRCA*-mutated individuals (Table 1).

**Table 1:** Results of PARP inhibitors in *BRCA*-mutated BC

Research	Study population	No. of patients	Study design	Regimen	Primary objective	Subgroup analysis		References
						Hormone receptor-positive	Triple-negative	
OlympiA	EBC with high risk of recurrence carrying germline *BRCA*1/2 mutations	1836	Phase III, double-blinded, randomized	Olaparib vs placebo	3 year iDFS: 85.9% vs 77.1% (HR = 0.58, *P* < 0.001)	3 year iDFS: 83.5% vs 77.2% (HR = 0.70, *P* = NA)	3 year iDFS: 86.1% vs 76.9% (HR = 0.56, *P* = NA)	[67]
OlympiAD	ABC with germline *BRCA*1/2 mutations	302	Phase III, open label, randomized	Olaparib vs TCP	mPFS: 7 m vs 4.2 m (HR = 0.58, *P* < 0.001)	12-mPFS: 79.6% vs 63.3% (HR = 0.82, *P* = NA)	12-mPFS: 79.4% vs 83.3% (HR = 0.43, *P* = NA)	[68]
EMBRACA	ABC with germline *BRCA*1/2 mutations	431	Phase III, open label, randomized	Talazoparib vs TCP	mPFS: 8.6 m vs 5.6 m (HR = 0.54, *P* < 0.001)	42-mPFS: 55.9% (HR = 0.47, *P* = NA)	42-mPFS: 44.1% (HR = 0.60, *P* = NA)	[69]
BRAVO	ABC with germline *BRCA*1/2 mutations	154	Phase III, open label, randomized	Niraparib vs TCP	mPFS: 4.1 m vs 3.1 m (HR = 0.96, *P*= 0.86)	ORR: 39.1% vs 52.0%	ORR: 31.7% vs 8.7%	[70]
BGB-290-201	ABC with germline *BRCA*1/2 mutations	88	Phase II, open label	Pamiparib	–	ORR: 61.9%	ORR: 38.2%	[71]
BROCADE3	ABC with germline *BRCA*1/2 mutations	509	Phase III, double-blinded, randomized	Veliparib + PCb vs placebo + PCb	mPFS: 14.5 m vs 12.6 m (*P* = 0.0016)	mPFS: 13.0 m vs 12.5 m (HR = 0.69, P = NA)	mPFS: 16.6 m vs 14.1 m (HR = 0.72, *P* = NA)	[72]

*EBC* early breast cancer, *ABC* advanced breast cancer, *TCP* standard therapy chosen by physicians, *PCb* paclitaxel + carboplatin, *m* month, *HR* hazard ratio, *mPFS* median progression-free survival, *NA* not available, *ORR* objective response rate

### Olaparib

A randomized, open-label, phase III study (OlympiAD) [68] compared the efficacy of olaparib monotherapy with that of physician’s choice of monotherapy in patients with a germline *BRCA1*/2-mutated and HER2-negative metastatic BC, with progression-free survival (PFS) as the primary endpoint. The median PFS in the olaparib group was significantly longer than in the standard chemotherapy group (7.0 months vs. 4.2 months; HR = 0.58, P < 0.001). At the 12 month follow-up, the PFS rates in the hormone receptor-positive individuals were 79.6% for the olaparib group and 63.3% for the standard therapy group, respectively (HR = 0.82, 95% CI 0.55–1.26). Meanwhile, the PFS rates in the triple-negative breast cancer (TNBC) patients were 79.4% for the olaparib group and 83.3% for the standard therapy group, respectively (HR = 0.43, 95% CI  0.29–0.63). The ORR for olaparib and standard therapy was 65.4% and 36.4% in the hormone receptor-positive subgroup and were 54.7% and 21.2% in the TNBC subgroup, respectively. In the OlympiAD study [73], the final OS did not significantly differ between patients treated with olaparib and with standard treatment in the ER-positive subgroup (median: 21.8 months for olaparib vs 21.3 months for standard therapy, HR = 0.86, 95% CI 0.55–1.36, *P* = NS). In the TNBC individuals, the median OS was 17.4 months for patients treated with olaparib and 14.9 months for patients treated with standard therapy (HR = 0.93, 95% CI 0.62–1.43, *P *= NS).

The OlympiA trial was a randomized, double-blind, phase III study of patients with HER2-negative, *BRCA1*/2 germline pathogenic or likely pathogenic variants and a high risk of recurrence of early BC [67]. The patients were randomized in a 1:1 ratio to oral olaparib or placebo for 1 year after surgery, (neo) adjuvant chemotherapy, and radiotherapy. The primary endpoint was iDFS. The 3 year iDFS rate was 85.9% in the olaparib group and 77.1% in the placebo group (HR = 0.58, *P *< 0.001). Subgroup analysis showed that the 3 year iDFS rate was 83.5% and 77.2% in the hormone receptor-positive group and 86.1% and 76.9% in the TNBC group, respectively. Based on the trial results, the Food and Drug Administration in March 2022 approved olaparib as adjuvant intensive therapy for the treatment of *gBRCA*-mutated, HER2-negative, recurrent high-risk early BC after surgery.

### Talazoparib

EMBRACA was a randomized, open-label, phase III study in which patients with HER2-negative, g*BRCA*-mutated advanced BC were randomized in a 2:1 ratio to talazoparib (1 mg daily) or physician’s choice of standard monotherapy (capecitabine, eribulin, gemcitabine, or vinorelbine, 21 days/cycle) [69]. The primary endpoint was PFS. The median PFS was significantly longer in the talazoparib group than in the physician’s choice group (8.6 months vs 5.6 months; HR = 0.54, *P* < 0.001). The ORR was higher in the talazoparib group than in the standard monotherapy group (62.6% vs 27.2%; OR = 5.0, 95% CI 2.9–8.8, *P* < 0.001). The 42-month PFS rates in the hormone receptor-positive group was 55.9% (HR = 0.47, 95% CI 0.32–0.71) and in the TNBC group was 44.1% (HR = 0.60, 95% CI 0.41–0.87). The ORR of the hormone receptor-positive subgroup was higher than that of the standard monotherapy group (63.2% vs 15.8%; OR = 2.89, 95% CI  1.43–5.83). The ORR of the TNBC group was also higher than that of the standard monotherapy group (61.8% vs 12.5%; OR = 11.89, 95% CI 4.54–41.37). These data show a clear benefit of talazoparib over chemotherapy, regardless of ER status. The final OS results of the EMBRACA trial showed that talazoparib has no superior survival benefit over chemotherapy (median OS: 19.3 months vs 19.5 months; HR = 0.848, 95% CI  0.670–1.073, *P* = 0.17) [74]. The OS rates for talazoparib and chemotherapy in the hormone receptor-positive group were 27.4% and 27.4% (HR = 0.827, 95% CI  0.597–1.143), respectively, and were 21.5% and 21.7% (HR = 0.899, 95% CI  0.634–1.276) in the TNBC group, respectively. In the chemotherapy arm, OS and total treatment time were shorter in patients who did not take subsequent PARP inhibitors or platinum treatment than in those who did. This indicates that subsequent therapy could narrow the OS difference between the two groups.

### Veliparib

BROCADE3 is a randomized, double-blind, phase III study that involved 509 patients (337 patients in the veliparib group and 172 patients in the control group) with *BRCA1/2* germline-mutated, HER2-negative advanced BC who had received up to two previous lines of chemotherapy for metastatic disease [72]. These patients were randomized in a 2:1 ratio to veliparib or placebo combined with PC chemotherapy (carboplatin and paclitaxel), and the primary endpoint was investigator-assessed PFS. The median PFS was longer in the veliparib group than in the control group (14.5 months vs 12.6 months, HR = 0.71, 95% CI 0.57–0.88, P = 0.0016). Meanwhile, the median OS tended to be longer in the veliparib group than in the control group (33.5 months vs 28.2 months; HR = 0.95, 95% CI 0.73–1.23, *P* = 0.67). In the hormone receptor-positive subgroup, the median PFS for veliparib and control treatment were 13.0 months and 12.5 months, respectively (HR = 0.69, 95% CI 0.52–0.92). In the TNBC group, the median PFS for veliparib and control treatment were 16.6 months and 14.1 months, respectively (HR = 0.72, 95% CI  0.52–1.01). Subgroup analyses showed comparable PFS benefits between hormone receptor-positive and triple-negative disease.

The subgroup analysis [75] results of the BROCADE3 study demonstrated that the 2- and 3- year PFS rates were 27.5% and 17.5%, respectively, in the hormone receptor-positive group and were 40.4% and 35.3%, respectively, in the TNBC group. Compared with placebo treatment, veliparib treatment achieved higher median OS (hormone receptor-positive group: 32.4 months vs 27.1 months, HR = 0.96, 95% CI 0.68–1.36, *P* = 0.832; TNBC group: 35.0 months vs 30.0 months, HR = 0.92, 95% CI 0.62–1.36, *P* = 0.683). These data indicate that the efficiency of veliparib in combination with chemotherapy does not vary with hormone receptor status.

### Pamiparib

In an open-label Chinese phase II study (BGB-290-201) [71], 88 patients with locally advanced or metastatic BC with deleterious or suspected deleterious mutations in *gBRCA1*/2 were treated with pamiparib 60 mg twice daily for 28 days per cycle. The primary endpoint was ORR. The secondary endpoints were duration of response (DOR), PFS, and OS. In the hormone receptor-positive subgroup, the ORR, median DOR, and median PFS were 61.9%, 7.49 months, and 9.2 months, respectively, and the median OS was not temporarily reached. In the TNBC subgroup, the ORR, median DOR, median PFS, and median OS were 38.2%, 6.97 months, 5.49 months, and 17.08 months, respectively. These findings supported the favorable efficacy of pamiparib in the hormone-positive group. Collectively, the above studies have shown a high clinical benefit of PARP inhibitors in ER-positive, *BRCA1*/2-mutated BC, improving the survival time of patients with metastatic BC.

## PARP inhibitor combined with immunotherapy

PARP inhibitor-mediated DNA damage fragments regulate the tumor immune microenvironment through a series of molecular and cellular mechanisms, including increased genomic instability, immune pathway activation, and cancer cell programmed cell death protein 1 expression. This may facilitate responses to ICIs [76]. Therefore, PARP inhibitors combined with ICIs therapy may have synergistic effects. The TOPACIO study used niraparib combined with pembrolizumab in the treatment of advanced triple-negative BC and found an ORR of 60% in *BRCA* somatic-mutated individuals [77]. The MEDIOLA study was an open-label, phase II trial that further explored the combining of PARP inhibitors and immunotherapy (i.e., olaparib plus durvalumab) for g*BRCA*-mutated advanced BC [13]. The 12-week disease control rate, as the primary endpoint, was 80%, and the ORR and median PFS were 63.3% and 8.2 months, respectively. In the subgroup analysis, the ORR and median PFS were significantly better in the hormone receptor-positive group (69.2% and 9.9 months, respectively) than in the TNBC group (58.8% and 4.9 months, respectively).

Compared with *BRCA1*-deficient tumors, *BRCA2*-deficient tumors have a greater abundance of genes expressing innate and acquired immunity and a greater population of macrophages, natural killer cells, T cells, and dendritic cells in the tumor microenvironment. Experiments have confirmed that *BRCA2*-deficient BC cells have higher responses to ICIs and significantly slower growth than *BRCA2*-deficient BC cells [78]. Therefore, the combination of PARP inhibitors and immunotherapy may have a significant effect in *BRCA2*-mutated BC, but more evidence-based research is needed.

## CDK4/6 inhibitor therapy

**Fig. 2:**
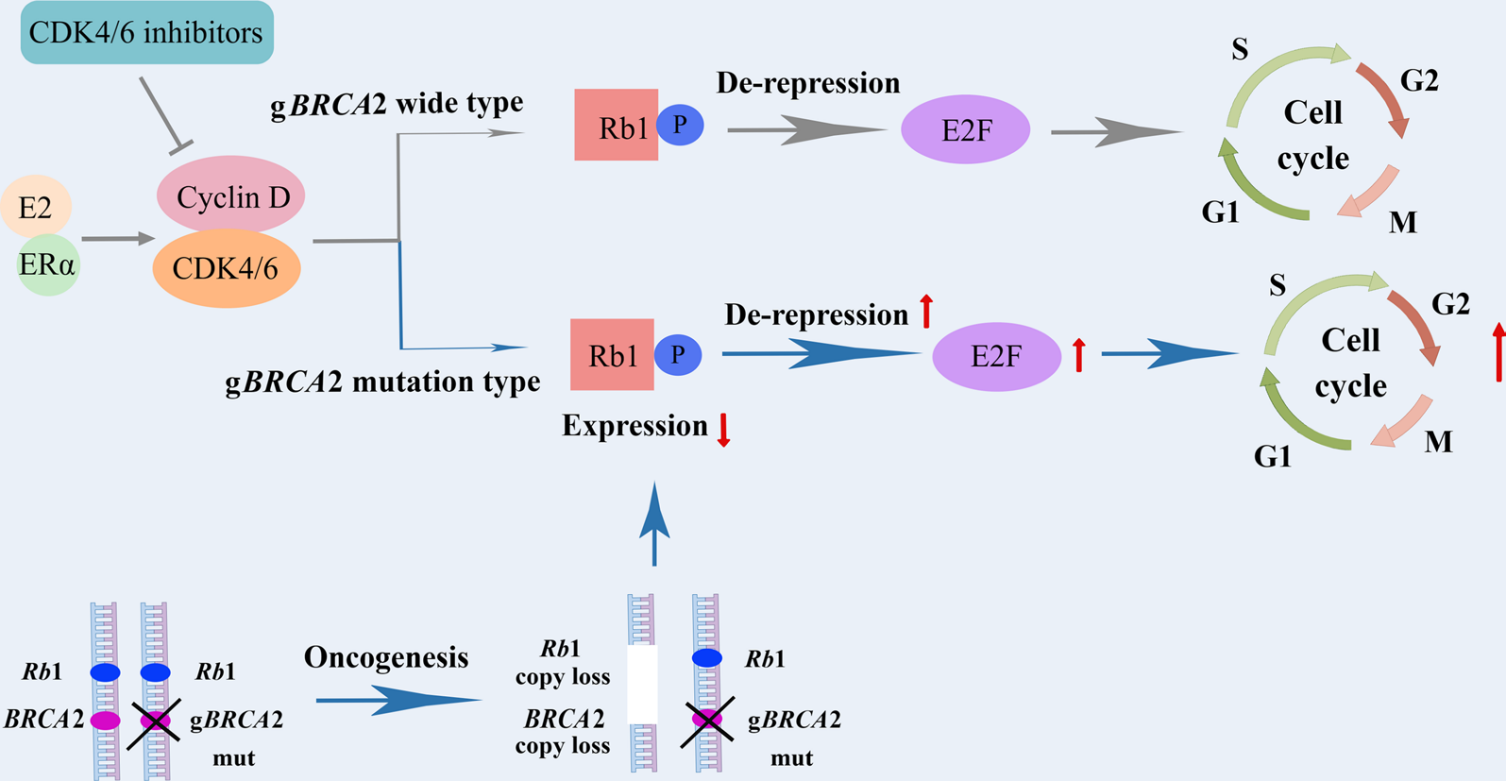
Resistance of CDK4/6 inhibitors induced by *gBRCA2* mutation in BC: In *gBRCA2* wild type BC cells, E2 binding to ERα promotes CDK4/6 to form a complex with Cyclin D, which is followed by phosphorylation of Rb1 and release of the transcription factor E2F. Then E2F promotes the cell cycle from G1 mitosis into S phase, leading to DNA replication [80]. In *gBRCA2* mutation BC cells, this is often accompanied by loss of *Rb1*, and reduced inhibition of E2F by Rb1, which promotes cell cycle progression. Rb1 deletion leads to the loss of downstream targets of CDK4/6, resulting in resistance to CDK4/6 inhibitors in *gBRCA2*-mutated BC [81, 82].

The combination of CDK4/6 inhibitors and endocrine therapy has become the standard first-line regimen for ER-positive/HER2-negative metastatic BC [79]. Given that both *BRCA2* and *Rb1* genes are located on chromosome 13q, loss of heterozygosity in *BRCA2* is common in BC with *BRCA2* germline mutation. Thus, concomitant *Rb1* deletions occur frequently in g*BRCA2*-mutated BC. Considering that Rb1 is a negative regulator of CDK4/6 pathway and that loss of *Rb1* leads to CDK4/6 inhibitor resistance, g*BRCA2*-mutated BC often exhibits endocrine plus CDK4/6 inhibitor resistance (Fig. 2). In the 2021 San Antonio Breast Cancer Conference (SABCS), a report from the Memorial Sloan Kettering Cancer Center showed that the median PFS of first-line CDK4/6 inhibitor combined with endocrine treatment for *gBRCA2*-mutated metastatic BC was shorter than that for *gBRCA2* wild type BC (7.0 months vs 14.7 months; HR = 2.32, 95% CI  1.38–3.91, *P* < 0.05). Among the individuals treated with CDK4/6 inhibitors, the median PFS was also significantly shorter in *gBRCA2* mutation individuals than in *gBRCA2* wild type individuals (4.4 months vs 10.2 months; HR = 2.12, 95% CI  1.48–3.03, *P* < 0.05).

Therefore, among ER-positive patients treated with adjuvant therapy or advanced therapy, CDK4/6 inhibitors may be ineffective for those with *BRCA2* germline mutations. PARP inhibitors can be used as the first choice for these patients.

## Conclusions

There is a regulatory relationship between ER and BRCA2. Increasing evidence supports that ER-positive, *BRCA2*-mutated BC is a special subgroup of ER-positive BC with poor prognosis and is fundamentally different from sporadic ER-positive BC. *BRCA2*-mutated BC patients have a higher risk of recurrence after surgery compared with sporadic BC patients. The choice of surgical methods can be based on the patient’s wishes provided that both advantages and disadvantages of BCS are adequately explained. BCS may be a relative contraindication for *BRCA2*-mutated BC. If a patient with a *BRCA2* mutation is willing to undergo BCS and is suitable for the procedure, BCS can be carefully chosen after informing them of the risk of ipsilateral BC recurrences. A PARP inhibitor is preferable to a CDK4/6 inhibitor in the adjuvant endocrine intensification strategy after surgery for ER-positive, *BRCA2*-mutated BC. The clinical value of PARP inhibitors combined with immunotherapy in the treatment of ER-positive and *BRCA2*-mutated BC needs to be verified in large-scale clinical studies. Genetic testing of newly diagnosed ER-positive BC is necessary to better guide treatment strategies and improve prognosis.

## Data Availability

Not applicable.

## Abbreviations

BC: Breast cancer
ER: Estrogen receptor
HER2: Human epidermal receptor 2
CDK4/6: Cyclin-dependent kinases 4 and 6
iDFS: Invasive disease-free survival
PARP: Poly (adenosine diphosphate-ribose) polymerase
ICIs: Immune checkpoint inhibitors
E2: Estradiol
SP1: Sequence-specific DNA binding proteins 1
OC: Ovarian cancer
PBM: Prophylactic bilateral mastectomy
PBSO: Prophylactic bilateral salpingo-oophorectomy
CBC: Contralateral breast cancer
AIs: Aromatase inhibitors
DFS: Disease-free survival
DSS: Disease-specific survival
DSB: Double-strand breaks
BCS: Breast-conserving surgery
IBTR: Ipsilateral breast tumor recurrence
NP: New primary tumor
TR: True local recurrence
BCT: Breast-conserving therapy
BCSS: Breast cancer-specific survival
OS: Overall survival
PFS: Progression-free survival
TNBC: Triple-negative breast cancer
ORR: Overall response rate
DOR: Duration of response
